# Enhancing the transformative potential of sustainability innovations: An application of the values-rules-knowledge framework

**DOI:** 10.1007/s13280-025-02148-2

**Published:** 2025-03-13

**Authors:** Caroline Hélène Dabard, Carsten Mann, Berta Martín-López

**Affiliations:** 1 Social-Ecological Systems Institute, Leuphana University Lüneburg, Universitätsallee 1, 21335 Lüneburg, Germany; 2 Eberswalde University for Sustainable Development, Schicklerstr. 5, 16225 Eberswalde, Germany

**Keywords:** Amplification, Decision contexts, Leverage points, Social innovation, Sustainability transformations, Values

## Abstract

To respond to global sustainability challenges with transformative solutions, there is a need to pinpoint the necessary and sufficient conditions that enhance the transformative potential of sustainability innovations. To this end, we examined 129 sustainability innovations in two European Biosphere Reserves, and analysed (1) their transformative potential, assessed based on a leverage points perspective, and (2) their supportive conditions (i.e. decision contexts, or constellations of values, rules and knowledge). Specifically, we used social network analyses to characterise different rules, or governance arrangements in the two Biosphere Reserves. By comparing the decision contexts of transformative and incremental innovations, we provide empirical evidence that plural values, coproduction and networks that are diverse, collaborative and influential, enable transformative innovations. Shallow leverage points seem insufficient but necessary to operationalise transformative change. Future research should explore the co-evolution of decision contexts and transformative potential, to better understand how to shift incremental to transformative innovations.

**Supplementary Information:**

The online version contains supplementary material available at 10.1007/s13280-025-02148-2.

## Introduction

Sustainability transformations are increasingly being acknowledged as necessary steps to ensure humanity’s future. However, such buzzwords like sustainability, innovation and transformations have been used as empty words and described as “blah blah blah” (Bentz et al. 2022), as they too often refer to incremental change, which reinforces existing problematic systems rather than challenges the status quo. Against this backdrop, there is a growing call in sustainability science to explore the characteristics of, and conditions for radical transformations, as opposed to incremental change (Temper et al. 2018; Feola et al. 2021).

Sustainability transformations encompass a variety of multi-scalar and multi-actor change processes that ideally seek to radically challenge problematic systems, and to foster justice and equity, as much as social-ecological integrity (Hölscher et al. 2018; Bennett et al. 2021; Bentz et al. 2022). However, only recently did scholars address the need to distinguish transformative from incremental change (Blythe et al. 2018; Feola et al. 2021; Rutting et al. 2022). In fact, to our knowledge, only a few empirical studies specifically assess transformative (versus incremental) impacts by researching, e.g. the contributions of initiatives to the Sustainable Development Goals (Jiménez-Aceituno et al. 2020), the impact of nature-based solutions (Palomo et al. 2021; Dubo et al. 2023; Goodwin et al. 2023), or the amplifying strategies adopted by sustainability projects (Tuckey et al. 2023). Dabard et al. (2024a) empirically assessed the transformative potential of multiple sustainability innovations by combining a leverage points perspective (Abson et al. 2017), with a typology of amplification processes (Lam et al. 2020). Yet, the transformative, or disruptive, character of studied interventions often remains taken for granted (Salomaa et al. 2020; Feola et al. 2021).

To better understand transformative innovations as opposed to incremental innovations, and how to foster them, there is a need to characterise their supportive conditions. There has been increasing knowledge of supportive conditions for sustainability transformations and innovations (e.g. Asheim et al. 2011, Biggs et al. 2012; Loorbach et al. 2017). Yet, without distinguishing transformative from incremental innovations, there is a risk to miss out on the specific supportive conditions that enhance transformative potential. Therefore, there is a need to provide empirical evidence of specific supportive conditions to transformative vs. incremental sustainability innovations. To achieve this, there is also a methodological need to decouple the analyses of supportive conditions (e.g. the contextual conditions, actors’ characteristics, and innovation processes) from the analysis of outcomes and transformative potential.

To target this gap, this study aims to identify the supportive conditions that enable transformative, rather than incremental, sustainability innovations. For this purpose, we examined 129 place-based sustainability innovations in two Biosphere Reserves (Dabard et al. 2024a). To identify their specific supportive conditions, we decoupled the analyses of conditions and outcomes of innovations as follows. To analyse supportive conditions, we examined the decision contexts, or systems of values, rules and knowledge (Gorddard et al. 2016) underpinning the development of these sustainability innovations. To analyse the outcomes, we assessed the transformative potential of the sustainability innovations through, (1) the impacts they produced according to a leverage points perspective (Meadows 1999; Abson et al. 2017), and (2) the amplifying strategies they implemented to increase their impacts (Lam et al. 2020). This allows us to compare the specific decision contexts underlying sustainability innovations archetypes of different transformative potential (i.e. transformative or incremental innovations). While the empirical analysis of transformative potential, values and knowledge production was part of Dabard et al. (2024a), in this study, we focus on the decision context, particularly on eliciting the governance arrangements underlying the different sustainability innovation archetypes through a social network analysis.

## Theoretical framework

### Sustainability transformations and innovations

Following Patterson et al. (2017, p.2), sustainability transformations can be defined as “fundamental changes in structural, functional, relational, and cognitive aspects of socio-technical-ecological systems that lead to new patterns of interactions and outcomes”. While the term ‘transformation’ is often used to convey a sense of radical, societal, and large-scale changes, the term often remains quite metaphorical (Feola 2015) and gave way to conceptual contributions (Bennett et al. 2019; Turnhout et al. 2020; Fisher et al. 2022). In contrast, empirical studies—such as this one—have often adopted more small-scale, bounded and place-based approaches, to examine, for example, sustainability initiatives (Lam et al. 2021; Londres et al. 2023), seeds of good Anthropocene (Bennett et al. 2016; Jiménez-Aceituno et al. 2020), or sustainability innovations (Dabard et al. 2024a). In this article, to study transformative change, we focus on place-based sustainability innovations, which we define as new pathways to meet human needs and aspirations, that generate positive outcomes for social-ecological integrity and equity (Dabard and Mann 2022). We understand place-based sustainability innovations as small-scale changes in practices, behaviours and ideas, which offer a bounded and managerial scale for analysis in comparison to greater societal transformation processes (Hölscher et al. 2018).

Scholars have increasingly pointed to the need for transformations to enhance both equity and sustainability (Leach et al. 2018; Bennett et al. 2021). Here, we follow a social-ecological perspective on sustainability and consider that sustainability innovations aim to enhance social-ecological integrity, or the long-term maintenance of social-ecological life support systems while intending to foster justice (Gibson 2006; Leach et al. 2018). We acknowledge that these are politized concepts with different meanings and implications to various people (Leach et al. 2018; Turnhout 2024) and, therefore, in this paper, we relied on the perspectives of the studied sustainability innovations to appraise what can be considered as positive, fair and sustainable outcomes.

We acknowledge that sustainability transformations, transitions, innovations, initiatives, or transformative change (and other related concepts) form a rich and diverse body of knowledge. For a more comprehensive overview of current debates and concepts, see, e.g. Feola (2015), Hölscher et al. (2018), Fisher et al. (2022). In this article, we focus specifically on sustainability innovations and we build on the frameworks of leverage points (Meadows 1999; Abson et al. 2017), amplifying strategies (Lam et al. 2020), and value-rules-knowledge (Gorddard et al. 2016; Colloff et al. 2017) to guide our empirical work.

### The transformative potential of sustainability innovations

Transformative potential is understood as the capacity to produce positive outcomes in terms of equity and social-ecological integrity, and to amplify positive change beyond the scope of innovation, thereby changing the social-ecological-technical systems where sustainability innovations are embedded (Dabard and Mann 2022; Dabard et al. 2024a).

In this study, to assess transformative potential, we build on a leverage points perspective to categorise outcomes (Meadows 1999; Abson et al. 2017), and on a typology of amplifying strategies (Lam et al. 2020). First, the leverage points perspective characterises interventions according to their capacity to shift a problematic system—from shallow leverage points that are easy to implement but with limited impact (for example slightly adapting how a system functions), to deep leverage points that are difficult to operationalise but have the capacity to shift systems (for example changing the underlying values and goals of a system) (Abson et al. 2017). Outcomes refer to the results and consequences of sustainability innovations, either expected or unexpected. We followed Gibson (2006) and Luederitz et al. (2017) to categorise sustainability outcomes into advances in: social-ecological integrity, intra- and intergenerational equity, livelihood sufficiency and opportunity, resource maintenance and efficient use, social-ecological stewardship and democratic governance, precaution and adaptation, structural physical and social changes. We then applied a leverage points perspective to differentiate between shallow and deep outcomes (see Dabard et al. 2024a). Second, we used the notion of amplifying strategies, that is, those actions and strategies that aim to scale the impacts of innovations beyond their own scope (Lam et al. 2020). We classified amplifying strategies as (1) amplifying within (e.g. enhancing, accelerating impacts within an innovations’ scope of action), (2) amplifying out (e.g. duplicating or transferring an innovation in another context) or (3) amplifying beyond and deep (e.g. changing the underlying values and the structure of a system) (Lam et al. 2020).

### Supportive conditions: the decision context framework

A recent and comprehensive analytical framework to appraise the conditions—or decision contexts—for transformative adaptation is the value-rules-knowledge framework (Gorddard et al. 2016; Colloff et al. 2017). According to this framework, decision contexts are defined as systems of specific values, rules and knowledge that shape, for example, transformative adaptation (Gorddard et al. 2016; Colloff et al. 2017; Lavorel et al. 2019), ecosystem management (Topp et al. 2022), or nature-based solutions (Dubo et al. 2023). Values refer to how people’s judgements regarding the importance of nature are justified in specific contexts, such as the transformative potential of innovations, and can be categorised as instrumental, intrinsic and relational (Chan et al. 2016; Pascual et al. 2023). Rules refer to formal and informal institutions, and to the governance arrangements that underlay transformative behaviour (Gorddard et al. 2016). Knowledge refers to different types of information, such as technical, scientific or experiential knowledge, and to the processes by which knowledge is produced by actors (Colloff et al. 2020).

The decision-context framework proved useful and comprehensive in recent studies, as it encompassed recent learnings about supportive conditions for innovation, resilience and transformation. For instance, transformation and resilience studies have shown that diverse networks and the participation of multiple actors foster radical and creative processes, notably because diverse actors would bring diverse values into such processes (Biggs et al. 2012; Avelino et al. 2017). Furthermore, innovations studies showed how multi-scalar governance arrangements may enable or hinder sustainability innovations (Bergek et al. 2015; Geels 2019). Finally, learning and experimenting, or participative knowledge coproduction, have been shown to be crucial to promote sustainability innovations (Wittmayer and Schäpke 2014; Chambers et al. 2022; Tuckey et al. 2023).

## Materials and methods

### Case studies

**Fig. 1:**
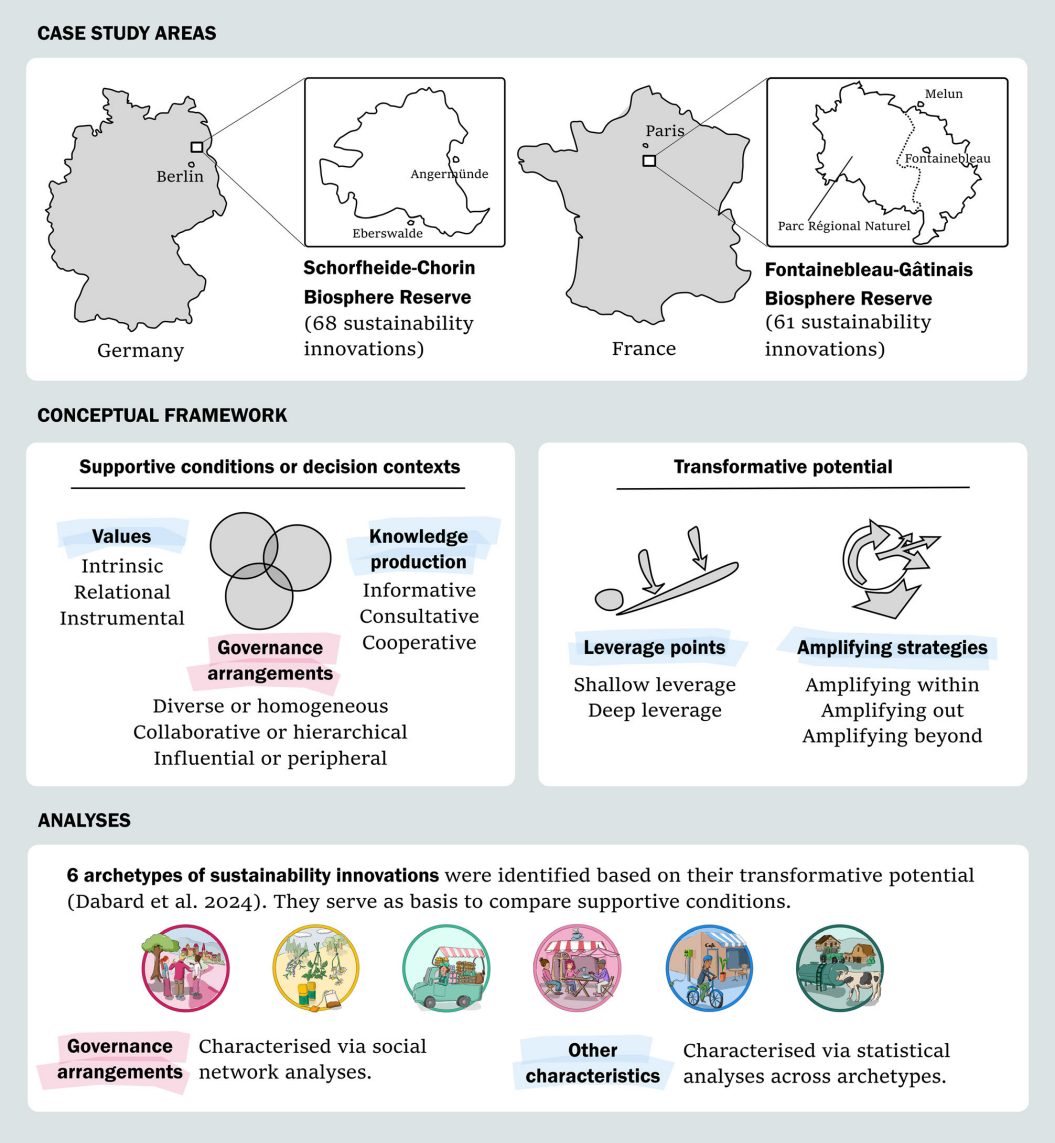
Methodological approach. The white boxes display (1) the location of the case studies, (2) the main components building our conceptual framework, distinguishing supportive conditions and transformative potential, and (3) the specific analyses conducted based on archetypes

Empirical data collection on sustainability innovations took place in two European Biosphere Reserves: Schorfheide-Chorin (Germany) and Fontainebleau-Gâtinais (France) (Fig. 1). Biosphere Reserves are model regions designated by UNESCO to showcase nature conservation, sustainable development as well as research, education and monitoring (UNESCO 2017). As such, Biosphere Reserves may arguably play the role of bridging organisations and foster learning, co-creation and transformative processes (Schultz and Lundholm 2011; Reed and Price 2020; Barraclough et al. 2021). In this regard, Biosphere Reserves are practical and compelling study sites to study sustainability innovations, as they have the capacity to foster innovative social networks and collaborative projects towards sustainable development (Dabard et al. 2024b, 2025).

Schorfheide-Chorin Biosphere Reserve is located approximatively 70 km north of Berlin and is now a well-established administrative unit with diverse projects regarding nature conservation, large-scale organic farming, tourism and education (UNESCO 2021b). Belonging to the State Office for Environment of the Brandenburg Ministry of Rural Development, Environment and Agriculture, the Biosphere Reserve administration is part of various horizontal and vertical planning and regulatory processes in several policy sectors. These cover agricultural and forestry land-uses, nature conservation, rural development, energy and urban planning. The administration is thus influenced by multi-sector and multi-level governance arrangements—and collaborates with various public and private actors and organisations such as two counties (in German *Landkreise*), the regional planning authority, local towns and city halls but also with private actors, NGOs, universities and research organisations.

Fontainebleau-Gâtinais Biosphere Reserve is located 60 km south of Paris. The Biosphere Reserve administration was created as an NGO, which board is composed notably by two counties (in French *départements*), the Région Île-de-France, the National Forest Office, the Regional Nature Park Gâtinais Francais, two universities, local environmental NGOs as well as local city halls (UNESCO 2021a). Although the associative management form of this Biosphere Reserve could enable participative and polycentric governance, the administrative structure proved challenging so far, because of the NGO's dependency on various institutions. Nonetheless, the Biosphere Reserve has implemented a few projects related to tourism and education. A large part of its territory is managed by the Parc Régional Naturel du Gâtinais Francais. Regional Nature Parks have similar missions to those of Biosphere Reserves in the French regulation (Mathevet and Cibien 2020). In comparison to the Biosphere Reserve, the Park has benefitted from considerable resources and implemented various projects with a strong focus on rural development through agro-ecology, tourism and mobility.

### Data collection

Semi-structured interviews were conducted with 17 regional informants in each Biosphere Reserve to identify sustainability innovations. The interviews were followed by a survey with representatives of 68 and 61 sustainability innovations in SCBR and FGBR, respectively. The survey targeted (1) the characteristics of each sustainability innovation regarding involved actors, processes and transformative potential and (2) their social networks. The first part of the survey was used to identify and characterise archetypes of sustainability innovations, in particular in terms of novelty type, sustainability impacts, amplifying strategies, values and knowledge production (Box 1). The second part of the survey listed relevant actors and asked the respondents to indicate those with whom they had a connection (e.g. project partnership, funding, sharing resources, informal exchanges). Relevant actors included all actors involved in sustainability innovations and those public organisations, which were deemed particularly relevant for innovation in the region by the regional informants. The survey data about relationships between actors was complemented through online searches, e.g. in cases where many official project partners were involved. In this case, the official websites were used as complementary information about lead and partner organisations.

### Sustainability innovation archetypes

With the goal to compare the transformative potential of sustainability innovations and their respective decision contexts, we built on an archetype analysis conducted on 68 (SCBR) and 61 (FBGR) innovations (Dabard et al. 2024a) (Fig. 1). The archetype analysis used variables related to outcomes of sustainability innovations, in particular to (1) their sustainability impacts and novelty types, which we characterised as shallow or deep leverage points; and (2) their amplifying, or scaling, strategies to enhance their impacts beyond their own scope of action (Table 1). We identified five archetypes in each Biosphere Reserve, based on their outcomes (Dabard et al. 2024a). We interpreted four archetypes to be common in both case study areas: Social and Sustainable Entrepreneurs, Social Innovations, New Sectors for Social-Ecological Transformation, and Service Innovations (Box 1). In SCBR, we furthermore identified Participative Transformation Governance projects, and in FGBR, we identified Technological Efficiency Innovations (Box 1). A short description of the transformative potential of each archetype is presented in the results.

**Table 1:** Description and operationalisation of the transformative potential and of the decision contexts of sustainability innovation archetypes

Characteristics of sustainability innovations	Description
*1. Transformative potential*	The capacity of sustainability innovations to produce and scale transformative outcomes, assessed through leverage points and amplifying strategies
Leverage points	The capacity of sustainability innovations to address shallow or deep leverage points in targeted systems, i.e. to induce incremental or radical change (Meadows 1999; Abson et al. 2017; Riechers et al. 2022). The leverage points are captured through their sustainability impacts (Gibson 2006; Luederitz et al. 2017) and their novelty types, e.g. social innovation, product innovation. These were categorised based on survey responses and attributed to either shallow or deep leverage points
Amplifying strategies	Strategies implemented to scale activities and impacts within (growing and accelerating), out (transferring and duplicating) and beyond (changing rules, structures and values (Lam et al. 2020), based on survey responses
*2. Decision context*	The systems of values, rules and knowledge that shape sustainability innovations (Gorddard et al. 2016; Colloff et al. 2017), captured through survey responses and social network analysis
Values	The way in which innovative actors value relationships to nature and relationships between people mediated by nature, categorised as instrumental, intrinsic and/or relational values (Pascual et al. 2023), categorised based on open-ended questions from the survey about missions, goals, values and sustainability aspects of sustainability innovations
Knowledge production	Forms of knowledge production and decision-making in sustainability innovations, categorised as informative, consultative or cooperative processes, based on survey responses
Rules	Rules-in-use and rules-in-form that underlay specific governance arrangements among sustainability innovations (Colloff et al. 2017). Governance arrangements are assessed here through social network analysis, in particular through the diversity, connectedness and influence of sustainability innovations in networks

**Box 1:**
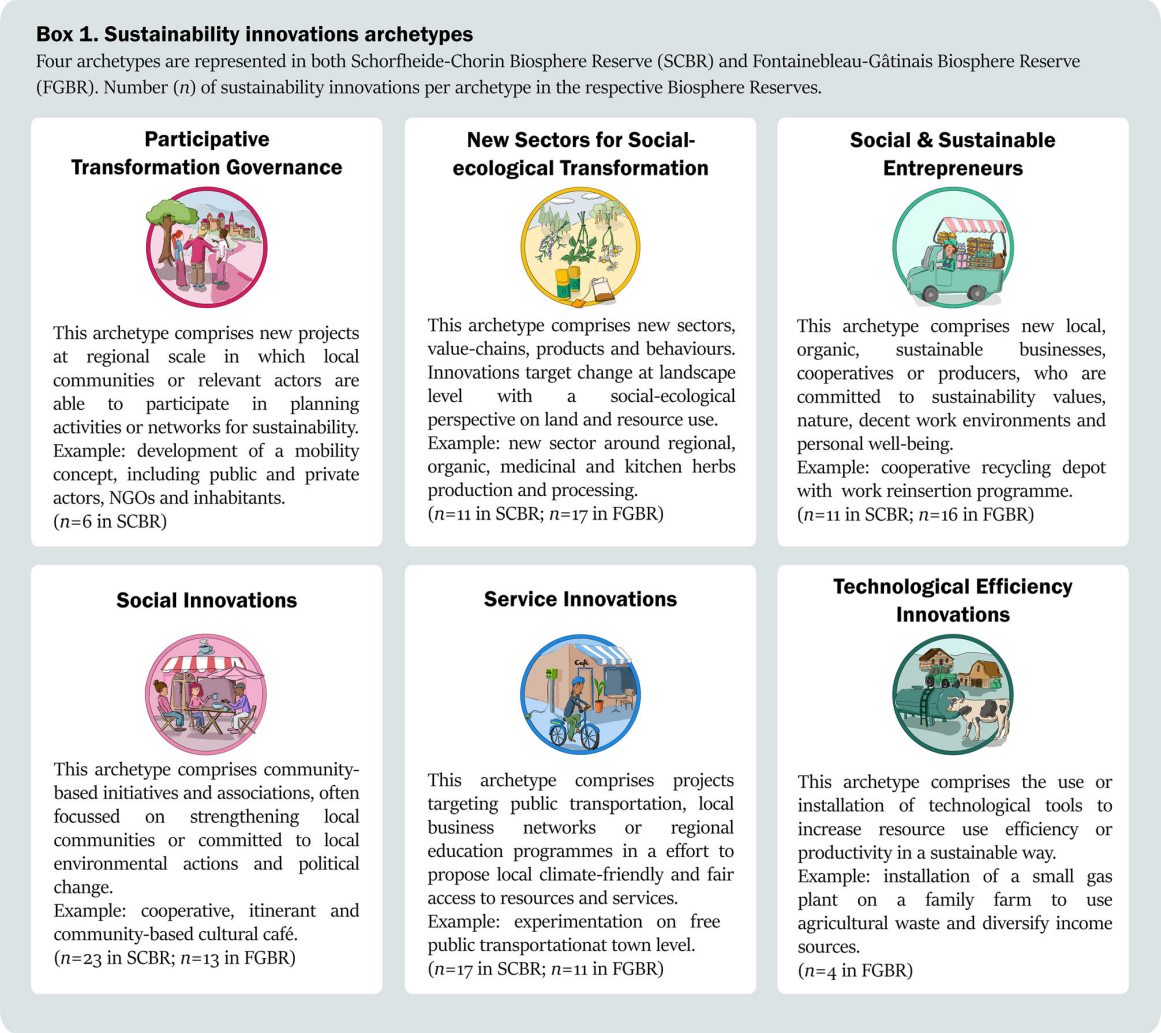
Sustainability innovations archetypes (Dabard et al. 2024a). The numbers in brackets indicate the number of innovations that were categorised within each archetype in both case study Biosphere Reserves. SCBR: Schorfheide-Chorin Biosphere Reserve; FGBR: Fontainebleau-Gâtinais Biosphere Reserve

### Values and knowledge production systems underpinning archetypes

We used survey data to characterise the archetypes in terms of (1) values and (2) knowledge production processes to assess the decision contexts of different sustainability innovations (Fig. 1, Table 1). First, we elicited values by categorising statements given by interviewees in response to open questions about their activities, their goals, missions and underlying values, and about the sustainable character of their innovation projects. Alike other empirical studies, we approached values by categorising the way in which innovative actors justified the importance of nature and sustainability within their innovation and decision-context, and coded the statements as expressing instrumental, intrinsic or relational values (Díaz et al. 2015, Chan et al. 2016; Topp et al. 2022). Instrumental values referred to the survey respondents expressing their motivation for their sustainability innovations, as based on their own benefit or the benefit of other human beings (Díaz et al. 2015). An example of an innovation’s decision-context based on instrumental values is a project on production of organic mushrooms within a cereal farm whose goal is to diversify income sources. Intrinsic values referred to a motivation of survey respondents for their projects based on, e.g. their belief that animals and nature should be regarded as ends in themselves, regardless of human use (Arias-Arévalo et al. 2017). An example was a conservation project that aimed to protect wildlife habitat for animal wellbeing. Finally, relational values referred to the motivation to enhance people’s relationships to nature or to enhance people-to-people relationships through nature and sustainability actions (Chan et al. 2016). An example of an innovation using relational values was the creation of community gardens as a means to enhance human-nature connectedness and to foster community-building.

We also collected data about knowledge production and the most common mode of decision-making within the innovation team, categorised as informative, consultative or cooperative. The survey asked the respondents to choose the most common mode of decision-making and collaborative deliberation. Informative deliberation and knowledge production referred to the project managers taking decisions and informing relevant partners and team members. Consultation referred to the project managers taking decisions after consulting relevant partners and team members. Cooperation referred to the project managers taking decisions together with relevant partners and team members. For full details about the different sustainability innovation archetypes and the archetype analysis, see Dabard et al. (2024a). We acknowledge that our approach to knowledge production could be strengthened by insights on the type of knowledge produced, such as technical, scientific or experiential knowledge (Lavorel et al. 2019; Topp et al. 2022; Dubo et al. 2023).

### Social network analysis and underlying governance arrangements

To complete the analysis of the decision contexts underlying sustainability innovation archetypes in terms of governance arrangements, we conducted a social network analysis (Fig. 1, Table 1). This approach to assess governance arrangements within innovative networks links to recent empirical studies making use of social network analysis to understand governance models for social-ecological systems resilience and transformation (e.g. Bodin and Tengö 2012; Salpeteur et al. 2017), or the diffusion and impacts of place-based innovations, initiatives or nature-based solutions (e.g. Feola and Butt 2017; Lam et al. 2021; Mitincu et al. 2023).

To compare governance arrangements, we created 12 social networks in total. Social networks represent nodes, in our case innovation actors, and edges, or connections between them. The nodes are categorised by: (1) archetype (whether the actors were involved in one, several or no archetypes), and (2) actor type (i.e. whether the actors were public organisations, private organisations, NGOs, civil society organisations or research organisations). We created full networks in each Biosphere Reserve which comprised all actors (*n* = 223 in SCBR; *n* = 305 in FGBR). Moreover, we created five archetype networks in each Biosphere Reserve, including only those actors directly involved in the corresponding sustainability innovation archetypes. These actors comprised the official project partners, funding organisations and those actors considered particularly important by the survey respondents. In all 12 networks, we computed actor centrality metrics, i.e. degree, betweenness and eigenvector. Degree centrality sums up each actor’s connections and thus shows how well connected one actor is. Betweenness centrality calculates how often an actor is on the shortest path between two others and thus shows how central, or bridging, an actor is. Eigenvector centrality computes how well connected an actor is to other important actors and therefore shows how influential an actor is. Specifically, eigenvector centrality is often used to show potential future influence (Prell 2015).

To articulate the governance arrangements which underly different archetypes, we characterised the social networks as: (1) diverse or homogeneous, (2) collaborative or hierarchical, and (3) influential or peripheral, through a series of statistical tests on centrality metrics in the full networks and in the archetype networks (Table 1). First, to address the diversity or homogeneity of different sustainability innovations, we tested for differences in the centrality metrics (degree and eigenvector) among actor types in each archetype network by applying Kruskal–Wallis test. We considered a network to be diverse when involving different actor types, here categorised as public, private, civil society, NGOs or research organisations. Second, to distinguish collaborative or hierarchical networks, we tested the betweenness centrality of different actor types in each archetype network. We considered networks to be collaborative when betweenness centrality was balanced across actor types, in opposition to hierarchical networks where one actor type dominated the others. Third, to assess the influence of archetypes, we tested for differences in the centrality metrics among the different archetypes in the full networks, using Kruskal-Wallis test. Furthermore, we performed Chi-square contingency tables and Fischer’s exact test to identify archetypes with a high representation of bridging actors, i.e. actors present in several archetypes and thus able to connect diverse actors and innovations. We acknowledge that our approach to characterise networks as diverse and collaborative is limited by the methods of social network analysis and could encompass other indicators of diversity and collaboration.

We do not present results for Technological Efficiency Innovations, because they were not significant, as their social network comprised only 11 actors. The social network visualisation and analyses were carried out using Gephi—an open-source software for exploring and manipulating networks and NodeXL. All statistical analyses were carried out using XLStat statistical and data analysis solution 2022 (Addinsoft, Paris, France).

## Results

In the following, the six identified archetypes, their transformative potential and decision contexts are presented, with a focus on governance arrangements through social network analysis.

**Fig. 2:**
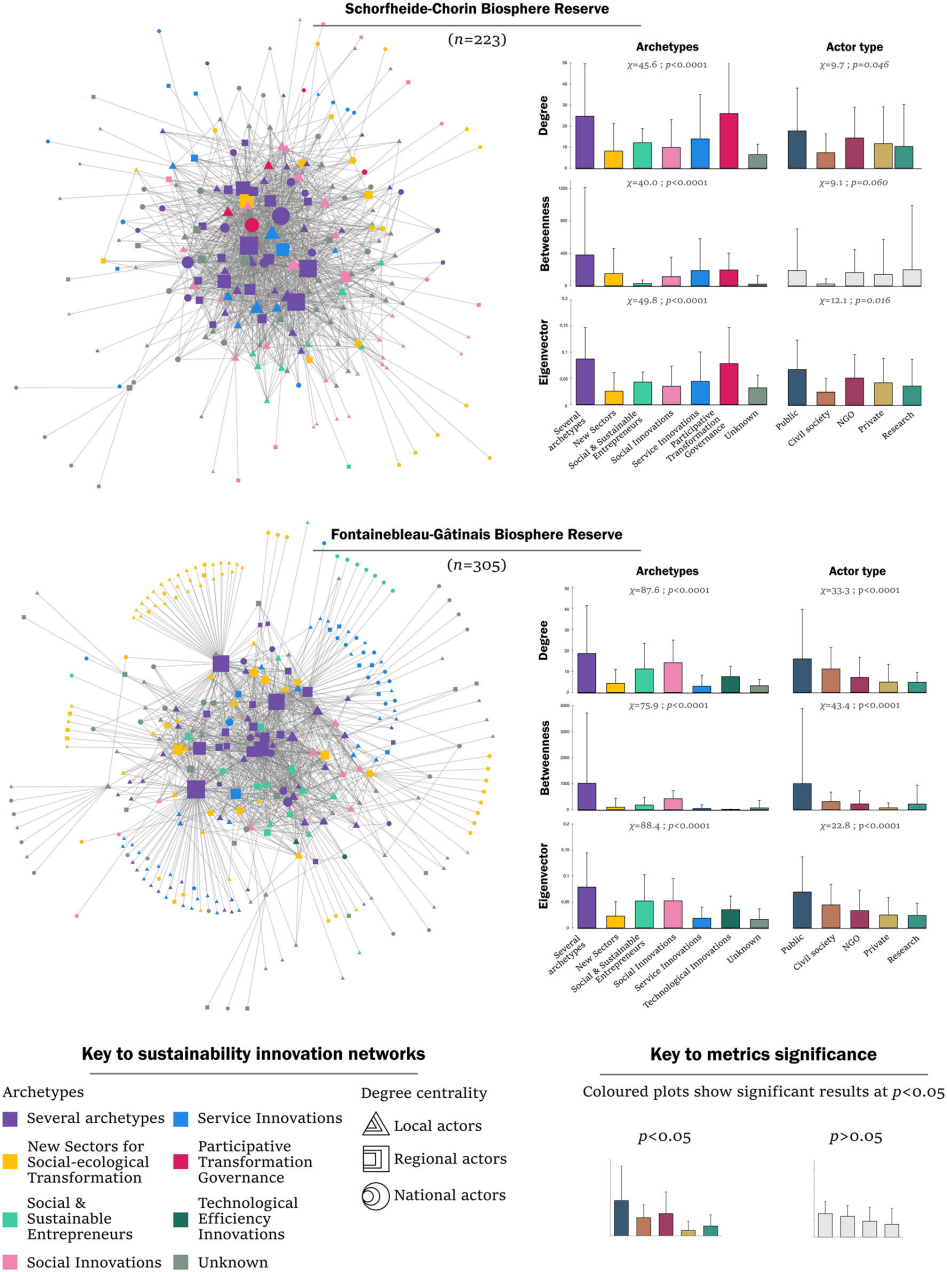
Social networks (left) in Schorfheide-Chorin Biosphere Reserve (top) and Fontainebleau-Gâtinais Biosphere Reserve (bottom), showing connections between all actors. Bar plots (right) show differences in centrality metrics (degree, betweenness, eigenvector) across archetypes and actor types

*Participative Transformation Governance.* This archetype, specific to SCBR, proved most transformative. The identified initiatives targeted deep and shallow leverages and implemented strong amplifying strategies to scale out (duplicating or transferring the innovation elsewhere), to scale beyond by changing people’s values (scaling deep) and by changing policy rules (scaling up) (Table 2). For instance, one project aimed to develop climate-friendly public transportation for the region, which meant reorganising the bus timelines to accommodate local people and tourists (shallow leverage point), but also collaborating with local towns, regional and national transportation firms and tourism actors to propose a holistic mobility concept across administrative units (deep leverage points). In Participative Transformation Governance, actors usually worked in a cooperative manner and reported plural values, i.e. instrumental, relational and intrinsic values. As for the governance arrangements underlying Participative Transformation Governance, we found that the networks involved a broad diversity of actors, who organised in a cooperative manner. The actor metrics in the archetype network revealed no significant differences in centrality (Fig. S1), suggesting that a diverse set of actors collaborated in a non-hierarchical manner. Furthermore, this archetype proved highly connected and influential, as was shown by the particularly high metrics of Participative Transformation Governance actors in the full SCBR network (Fig. 2) and by the high number of bridging actors, which are actors engaged in several archetypes, including in Participative Transformation Governance (Table S1).

**Table 2:** Characterisation of sustainability innovation archetypes in terms of transformative potential and decision context, defined by the values-rules-knowledge framework. The percentages indicate the most reported novelty types, sustainability impacts, values and knowledge production modes in the respective archetypes, based on survey data. Governance arrangements are defined according to the network analysis—specifically, how diverse or homogenous, collaborative or hierarchical and influential or peripheral each archetype was

Archetype	Transformative potential	Decision context
*Schorfheide-Chorin Biosphere Reserve*		
Participative Transformation Governance (*n* = 32)	**Deep leverage** Novelty type: new values (100%), organisation (67%) Impacts: social-ecological stewardship & democratic governance (83%), precaution & adaptation (67%) **Strong scaling potential** Amplifying: scaling out (83%) and beyond (83%)	**Values**: plural values (relational: 67%, intrinsic: 50%, instrumental: 33%) **Knowledge production**: cooperative (83%) **Governance arrangements:** Diverse, collaborative and influential network
New Sectors for Social-ecological Transformation (*n* = 60)	**Shallow & deep leverages** Novelty type: new sector (91%), behaviour (64%), technology (55%), product (27%) Impacts: social-ecological integrity (45%), precaution & adaptation (36%) **Limited scaling potential** Amplifying: scaling beyond (55%),	**Values**: instrumental (64%), intrinsic (36%) **Knowledge production**: consultative (45%) **Governance arrangements:** Diverse, collaborative and peripheral network
Social & Sustainable Entrepreneurs (*n* = 23)	**Deep leverage** Novelty type: new values (73%), service (64%) Impacts: N.A **Limited scaling potential** Amplifying: N.A	**Values**: relational (100%) **Knowledge production**: NA **Governance arrangements:** Homogenous, collaborative and peripheral network
Social Innovations (*n* = 89)	**Deep leverage** Novelty type: new organisation (70%) Impacts: structural social change (91%) **Limited scaling potential** Amplifying: N.A	**Values**: relational (87%) **Knowledge production**: cooperative (48%), consultative (43%) **Governance arrangements:** Diverse, hierarchical and peripheral network
Service Innovations (*n* = 56)	**Shallow leverage** Novelty type: new market (70%), new product (24%) Impacts: resource maintenance and efficient use (82%), structural physical change (71%) **Limited scaling potential** Amplifying: N.A	**Values**: instrumental (71%) **Knowledge production**: informative (29%) **Governance arrangements:** Diverse and collaborative, average influence
*Fontainebleau-Gâtinais Biosphere Reserve*		
New Sectors for Social-ecological Transformation (*n* = 120)	**Shallow & deep leverages** Novelty type: new organisation (88%), sector (29%), product (24%) Impacts: social-ecological integrity (35%) **Limited scaling potential** Amplifying: N.A	**Values**: relational (94%), instrumental (82%) **Knowledge production**: consultative (47%) **Governance arrangements:** Diverse, hierarchical and peripheral network
Social & Sustainable Entrepreneurs (*n* = 46)	**Shallow & deep leverages** Novelty type: new values (100%), service (88%), organisation (88%) Impacts: intra- & intergenerational equity (25%) **Deep scaling potential** Amplifying: scaling deep (69%)	**Values**: relational (100%), instrumental (56%), intrinsic (25%) **Knowledge production**: NA **Governance arrangements:** Diverse, collaborative and influential network
Social Innovations (*n* = 39)	**Deep leverage** Novelty type: new behaviour (54%), values (85%) Impacts: livelihood sufficiency & opportunity (38%) **Limited scaling potential** Amplifying: N.A	**Values**: relational (85%), instrumental (85%) **Knowledge production**: cooperative (62%) **Governance arrangements:** Diverse, collaborative and influential network
Service Innovations (*n* = 109)	**Shallow leverage** Novelty type: new service (64%) Impacts: N.A **Limited scaling potential** Amplifying: N.A	**Values**: relational (85%), instrumental (45%) **Knowledge production**: informative (45%) **Governance arrangements:** Diverse, hierarchical network and peripheral network

*New Sectors for Social-ecological Transformation.* In SCBR, New Sectors revealed disparate transformative outcomes (Table 2). Many sustainability innovations in this archetype targeted shallow and deep leverages, for example developing organic products (shallow leverage point) and collaborative, regional partnerships and value-chains in the agricultural sector (deep leverage point). Yet, New Sectors had limited scaling potential, with a lack of amplifying strategies. With regards to their decision context, New Sectors in SCBR relied on instrumental values, for example seeing organic agriculture as a means for regional development and job creation. Respondents reported about both consultative and collaborative knowledge production. As for the governance arrangements underlying New Sectors, we found a rather diverse, collaborative, but specialised network. We did not find significant differences in centrality metrics in the archetype network, showing that all actors collaborated at a similar level (Fig. S1). Likewise, we found that actors in the New Sectors did not act as bridging actors in the region (Table S1). In fact, they presented low centrality metrics in the full SCBR network (Fig. 1), suggesting a rather peripheral, very loosely connected archetype.

In FGBR, New Sectors for Social-ecological Transformation had a similarly disparate transformative potential, acting on both shallow and deep leverages, albeit with rare amplifying strategies (Table 2). For instance, some projects aimed to develop local and organic school canteens, which fostered consumption of seasonal vegetables (shallow leverage point). This also required the collaborative reorganisation of public catering with public organisations, local producers and caterers—with the motivation to foster healthy and local organic food—and to strengthen rural small businesses (deep leverage points). As to their decision context, New Sectors in FGBR built on mostly relational and instrumental values and a consultative knowledge production mode. The social networks revealed a hierarchical governance structure behind the New Sectors in FGBR, particularly influenced by public actors (Fig. S1). Indeed, the archetype network showed very strong differences in centrality metrics across actor types, with public actors dominating the network with high degree, betweenness and eigenvector centrality. Furthermore, New Sectors proved to be peripheral in FGBR, as shown by the particularly low centrality in the full FGBR network (Fig. 1) and the very few bridging actors that were involved in this archetype (35% of actors; Table S1). In fact, we found a positive significant association between the number of actors and being exclusively involved in the New Sectors archetype (Fischer’s exact test *p* = 0.005; Table S1).

*Social & Sustainable Entrepreneurs.* In SCBR, Entrepreneurs had a rather limited transformative potential. Although their actions focused on deep leverage points, such as raising awareness and thereby aiming to shift values and individual behaviour towards sustainability, their amplifying strategies were limited (Table 2). For example, a local nature tour guide aimed to foster human-nature connectedness by offering workshops and walking tours through nature, thereby raising awareness about various sustainability topics. Regarding the decision context, Entrepreneurs had strong relational values but no specific form of knowledge production, most likely because they involved very few actors. The social network analysis revealed a small, rather loose network of dispersed and homogenous actors (Fig. S3). Social and Sustainable Entrepreneurs showed no differences in centrality metrics across actor types, suggesting a collaborative network. However, this archetype was peripheral in the full SCBR network, with a particularly low betweenness centrality of Social and Sustainable Entrepreneurs (Fig. 2).

In FGBR, Social and Sustainable Entrepreneurs had more transformative impact. This archetype targeted both shallow and deep leverage points. For example, several grassroots organic cooperatives supported local farmers and sold organic products (shallow leverage point)—while also trying to build alternatives to capitalist consumption by fostering community-building and regional networks (deep leverage point). Some innovations reported strategies to scale beyond, most notably through shifting values and behaviours, for example regarding consumption (Table 2). Regarding the decision context, respondents in this archetype reported relational values, e.g. with a focus on community-building, while the knowledge production mode remained unspecified, mostly due to very small teams in this archetype. As for the governance arrangements related to this archetype, we found a diverse, collaborative, rather influential network influence. Rather balanced centrality metrics in the archetype network revealed that diverse actors collaborated on similar level (Fig. S3). In the full FGBR network, Entrepreneurs had a particularly high eigenvector centrality, suggesting that this archetype was well-connected to influential actors and could gain in influence in the future (Fig. 2).

*Social Innovations.* In SCBR, Social Innovations had disparate outcomes, with actions targeting deep leverage points, but limited scaling strategies (Table 2). For instance, a village cultural association aimed to foster lively rural communities through culture, and offered arts-based workshops to teenagers and children about human-nature relationships. The decision context underlying Social Innovations consisted of relational values and knowledge production in a cooperative or consultative manner. Surprisingly for Social Innovations, the network analysis revealed rather hierarchical governance arrangements. Indeed, the archetype network appeared quite dominated by one research organisation, with especially high centrality metrics (Fig. S4). Furthermore, this archetype appeared peripheral, very loosely connected to other innovations in the full SCBR network. Social Innovations had a very low centrality (Fig. 2) and particularly few bridging actors since we found a positive association between being actor of Social Innovation and being exclusively involved in this archetype (Fischer’s exact test *p* = 0.017; Table S1).

On the contrary, in FGBR, Social Innovations had a strong transformative potential, with actions targeting both shallow and deep leverage points. However, their reported amplifying strategies remained quite limited (Table 2). For instance, a local branch of a national citizen association for democracy pressured the local city hall and negotiated with election candidates to implement concrete sustainability measures at town level, for example for the city hall to purchase sustainable supplies (shallow leverage point), but also to create a local and independent long-term commission for nature protection (deep leverage point). Regarding their decision context, Social Innovations in FGBR relied on relational and instrumental values and they established processes of knowledge coproduction. The network analysis highlighted a rather diverse, collaborative network of high influence since Social Innovations actors had high centrality in the full FGBR network (Fig. 2) and were strongly involved in the other archetypes (Fischer’s exact test *p* < 0.001; Table S1). The archetype network showed no differences in betweenness centrality among different actor types, which revealed a collaborative working mode (Fig. S4).

*Service Innovations.* In SCBR, Service Innovations had a very limited transformative potential. They targeted shallow leverage points and rarely implemented amplifying strategies (Table 2). For example, a new service was developed for e-bike tourists: a network of places to re-load their bike and a labelling action as e-bike friendly region. The decision context relied on instrumental values and processes of knowledge production that are merely informative—and sometimes cooperative. The governance arrangements relied on a rather collaborative network, with diverse actors that had similar centrality metrics, suggesting cooperation at eye-level (Fig. S5). In the full SCBR network, Service Innovations had average centrality metrics, and therefore average influence (Fig. 2).

In FGBR, Service Innovations had a similarly low transformative potential, with actions targeting shallow leverage points and limited amplifying strategies. An example was the creation of product labels for local businesses and organisations that broadly aligned with the goals of the Parc Régional Naturel or of the Biosphere Reserve. Service Innovations relied mostly on relational values and processes of knowledge production that were informative (Table 2). The network analysis revealed a hierarchical and very loosely connected network. Indeed, Service Innovations had low centrality metrics in the full FGBR network (Fig. 2) and the archetype network showed that public actors were particularly influential in comparison to all other actor types (Fig. S5).

## Discussion

### Necessary conditions to enhance transformative potential: plural values, knowledge coproduction and diverse, collaborative, influential networks

This study provided empirical evidence of three necessary conditions to enhance transformative potential, along the decision context framework: (1) the existence of plural values, (2) knowledge coproduction, and (3) networks that are diverse, collaborative and influential. In contrast, incremental innovations were connected to instrumental values and hierarchical, homogenous and peripheral networks.

These results advance our understanding of supportive conditions for transformative innovations. So far, insights from transitions and innovations studies (Asheim and Coenen 2005; Wittmayer et al. 2018), as well as from resilience and transformations studies (Moore et al. 2012, Westley et al. 2013), have argued that diverse networks and collaborative partnerships were crucial for sustainability transitions and transformations. Our findings advance these earlier claims by providing empirical evidence that plural values, coproduction and networks that are diverse, collaborative and influence, are connected to transformative—rather than incremental—innovation.

Indeed, without decoupling the analysis of transformative potential from supportive conditions, there is a risk of missing evidence on the specific conditions that are required and necessary for transformative versus incremental innovations. So far, there has been limited evidence regarding supportive conditions and increase in transformative potential. One example study showed that plural values (including instrumental, intrinsic and relational), diverse knowledge systems and collaborative governance underlined transformative nature-based solutions with a higher capacity for transformative adaptation (Dubo et al. 2023). In contrast, instrumental values, top-down regulations and formal governance arrangements were connected to nature-based solutions with low capacity for transformative adaptation.

In this vein, by comparing supportive conditions assessed through the decision context framework and transformative potential, this study showed that the most transformative archetype, Participative Transformation Governance in SCBR, was characterised by plural values, knowledge coproduction and a diverse and influential network. Cooperative and diverse networks were also involved in innovations with mixed transformative potential, such as New Sectors for Social-ecological Transformations in SCRB – but their decision-context did not rely on plural values. This suggests that the existence of plural values underpinning the decision-contexts of innovation networks is a necessary condition to enhance the innovations’ transformative potentials. Similarly, Social and Sustainable Entrepreneurs in FGBR (mixed transformative potential), who also built on diverse, collaborative and influential networks, reported rather plural values – but no evidence of knowledge coproduction was found. This might be because Entrepreneurs mostly worked independently rather than in teams. In addition, incremental innovations reported hierarchical decision-making, therefore underlining that collaboration is another necessary condition to enhance transformative potential. By decoupling the analyses of transformative potential and of supportive conditions, this study thus provided novel empirical evidence about necessary conditions to enhance transformative potential.

### Shallow leverage points are insufficient—but necessary for transformative change

This study advances scholarship on leverage points as indicators of transformative potential (Riechers et al. 2022), by providing evidence that most transformative innovations build on both shallow and deep leverage points. We argue further that shallow leverage points, albeit insufficient for transformative change, are necessary, as was suggested in Evans et al. 2024.

Indeed, we observed that sustainability innovations, which focussed on deep leverage points, such as value and behaviour shifts, but which lacked concrete, more shallow outcomes, had limited transformative potential. For instance, Social Innovations in SCBR and Social and Sustainable Entrepreneurs in FGBR displayed mixed transformative potential despite focusing on deep leverage points, such as awareness-raising activities aiming to shift people’s worldviews and behaviours. Here, we argue that such innovations lack more concrete, easy-to-implement actions (shallow leverage points) that can pave the way for transformation. These results are in line with Burgos-Ayala et al. (2020), who showed that Columbian environmental management projects, which tackled only deep leverages, for example, those projects that re-design management based on Indigenous people’s values and worldviews, lacked concrete changes at shallower levels, jeopardizing their transformative potential.

Based on our results, we propose that shallow innovations, even though insufficient for transformative change, are necessary to operationalise transformative innovation by producing concrete, measurable and short-term impacts, by creating milestones for action and by providing opportunities for learning, network-building and co-creating innovation processes. However, how to foster chains of leverages, e.g. from shallow innovation paving the way for radical transformation, and how to disentangle interactions between leverages, remains to be further explored (Riechers et al. 2021, 2022).

### Enhancing transformative potential: the role of Biosphere Reserves

Our approach to assessing the decision context and transformative potential of sustainability innovations may support strategies to enhance their transformative outcomes. For instance, incremental innovations such as Service Innovations could enhance impacts by diversifying their actions towards more deep leverage points and by implementing amplifying strategies. For this purpose, diversifying their networks would prove useful, as this would foster collaboration and potentially bring in new ideas and goals – but also providing opportunities for scaling strategies (Biggs et al. 2012; Kratzer and Ammering 2019; Lam et al. 2021; Mitincu et al. 2023). Reversely, innovations that focus on shifting people’s worldviews and behaviours, such as Social and Sustainable Entrepreneurs or Social Innovations, could enhance their transformative outcomes by designing actions that target concrete, albeit shallow, leverage points and more concrete scaling strategies to transfer or duplicate. A possible avenue to operationalise their strong visions and relational values could be to build up or make use of existing partnerships with influential and incumbent actors, such as Biosphere Reserves and regional public organisations, which have the capacity to foster and broker innovations (Mitincu et al. 2023). In fact, we found that Biosphere Reserves, Regional Nature Parks, and other regional public administrations played a central role in the innovative networks as bridging organisations and were greatly involved in developing place-based sustainability innovations with strong transformative potential.

These results suggest that Biosphere Reserves can play a strong role in highlighting and actively supporting place-based sustainability innovations as model regions, thereby advancing actionable knowledge for sustainability transformations (Barraclough et al. 2021; Dabard et al. 2024b). In particular, we propose that Biosphere Reserves could purposefully support sustainability innovations by providing experimental spaces, increasing the visibility of existing initiatives and projects, and by strengthening regional networks among a diversity of actors, to broker knowledge and collaborations (Mitincu et al. 2023; Dabard et al. 2025). Here, it seems that defining fields of expertise and diversifying activities and partnerships can strengthen the role and impacts of Biosphere Reserves. Finally, we pledge for stronger commitment to sharing knowledge and experiences across boundaries of BRs, for example in the World Network of Biosphere Reserves (Dabard et al. 2024b, 2025). As similar sustainability innovations were found in our two case studies, such experiences are likely relevant in other Biosphere Reserves as well. Thus, there probably is a strong pool of knowledge about place-based initiatives and innovations in the World Network, which should ensure cross-boundary learning and inspiration (Barraclough et al. 2021). Such cross-boundary exchanges would strengthen Biosphere Reserves as worldwide model regions for sustainability transformations.

### Methodological limitations and future research

Sustainability innovations and transformations aim to enhance social-ecological integrity and to foster justice. They are politized concepts, which may be appraised differently, as positive or negative, by different actors. In this study, we took into account different potential outcomes of sustainability innovations, from enhancing equity to resource maintenance to social-ecological integrity (Dabard et al. 2024a). In this regard, the assessment of transformative potential was based on outcomes reported by respondents, who were representatives of sustainability innovations. The reported outcomes were categorised by the authors. Hence, it is important to note that we built on the understanding of the respondents to assess what were considered desirable and positive outcomes. As such, this approach gives space to the respondents’ potentially diverging views on desirable and positive sustainability innovations. Yet, we also acknowledge that it was beyond the scope of this study to collect and assess information on potentially diverging perspectives of, e.g. innovation insiders and outsiders regarding advances in terms of equity and social-ecological integrity. In this regard, future empirical research should engage with the underlying assumptions and diverging perspectives of different actors regarding what can be considered as positive and desirable outcomes of sustainability innovations and transformations. This would contribute to a fairer understanding of plural transformations pathways, based on diverse constellations of objectives and values in different contexts (Turnhout 2024).

Finally, the study built on data collected through one-time survey interviews and thus did not capture the probable co-evolution of decision contexts with transformative outcomes. It has been shown that values, rules and knowledge co-evolve (Lavorel et al. 2019; Dubo et al. 2023), and it is likely that they would influence and be influenced by transformative processes. For instance, it is likely that the way innovation actors value nature would evolve throughout innovative processes in partnership with diverse actors (Pascual et al. 2023). Moreover, networks and amplifying strategies are likely to co-evolve as well (Lam et al. 2021). Amplifying strategies push actors to seek new partnerships, and new partnerships enable further amplifying strategies. Future research should consider long-term data collection to capture the potential co-evolutions of conditions of innovation and their outcomes.

## Conclusion

By comparing the supportive conditions that fostered sustainability innovations of varying transformative potential, we identified three necessary conditions that enhance transformative potential: holding plural values, setting processes of knowledge coproduction, and developing diverse, cooperative and influential networks. By contrast, incremental innovations are based on instrumental values and hierarchical, homogeneous networks. These findings advance our understanding of necessary conditions for transformation by empirically differentiating between sustainability innovations that are incremental versus transformative ones. Furthermore, while actions targeting behavioural or value shifts (for example via education and awareness-raising) are still broadly considered as the main pathway towards radical, societal change (deep leverage points)—our results rather suggest that more concrete, short-term and easy-to-implement actions (for example new products, services, collective actions) are necessary for transformative innovations, because such practical steps foster learning, provide milestones and build up momentum (shallow leverage points). Yet, future research should identify strategies to shift incremental innovations to radical, deeply transformative change. Finally, this study suggests that Biosphere Reserve administrations and policy-makers can actively shape supportive conditions by: (1) connecting with diverse actors, (2) activating and nurturing plural values, (3) building on different types of knowledge and (4) strengthening collaborative decision-making.

## Supplementary Information

Below is the link to the electronic supplementary material.

Supplementary file1 (PDF 680 kb)
