# Supplementary material for: Enhancing the transformative potential of sustainability innovations: An application of the values-rules-knowledge framework
Source: Ambio. 2025 Mar 13;54(7):1250–66. doi: 10.1007/s13280-025-02148-2 (PMC12133668; doi:10.1007/s13280-025-02148-2)
Supplement: Supplementary file 1 — Supplementary file1 (PDF 680 kb) [file 13280_2025_2148_MOESM1_ESM.pdf]

Ambio

Supplementary Information

This supplementary information has not been peer reviewed.

Title:

Enhancing the transformative potential of sustainability innovations: an application of the values-rules-knowledge framework

Supplementary materials

Table S1. Number of actors (percentage presented in brackets) involved only in one archetype or in several archetypes and chi-squared test per Biosphere Reserve. * denote significant positive association at 5% significance level according to Fisher's exact test.

Sustainability innovations archetypes	Nb of actors involved only in the archetype (%)	Nb of actors involved in several archetypes (%)	Chi-square test
Schorfheide-Chorin Biosphere Reserve			χ^2=10.071, p=0.039
Participative Transformation Governance (n=32)	7 (22%)	25 (78%)*	
New Sectors for Social-ecological Transformation (n=60)	26 (43%)	34 (57%)	
Social & Sustainable Entrepreneurs (n=23)	8 (35%)	15 (65%)	
Social Innovations (n=89)	46 (52%)*	43 (48%)	
Service Innovations (n=56)	21 (36%)	37 (64%)	
Fontainebleau-Gâtinais Biosphere Reserve			χ^2=18.926, p<0.001
New Sectors for Social-ecological Transformation (n=120)	78 (65%)*	42 (35%)	
Social & Sustainable Entrepreneurs (n=46)	23 (50%)	23 (50%)	
Social Innovations (n=39)	10 (26%)	29 (74%)*	
Service Innovations (n=109)	60 (56%)	47 (44%)	

Table S2. Network properties of full networks and archetype networks of the Schorfheide-Chorin Biosphere Reserve (SC) and Fontainebleau-Gâtinais Biosphere Reserve (FC).

	Full network		New Sectors for Social-ecological Transformation		Social & Sustainable Entrepreneurs		Social Innovations		Service Innovations		Participative Transformation Governance	Technological Efficiency Innovations
Biosphere Reserve	SC	FG	SC	FG	SC	FG	SC	FG	SC	FG	SC	FG
Number of nodes	223	305	60	120	23	46	89	39	56	109	32	11
Graph density	0.06	0.02	0.14	0.05	0.17	0.18	0.12	0.19	0.22	0.04	0.28	0.35
Average geodesic distance	2.43	2.8	2.16	2.51	2.19	2.12	2.17	2.07	1.99	2.31	1.72	1.65

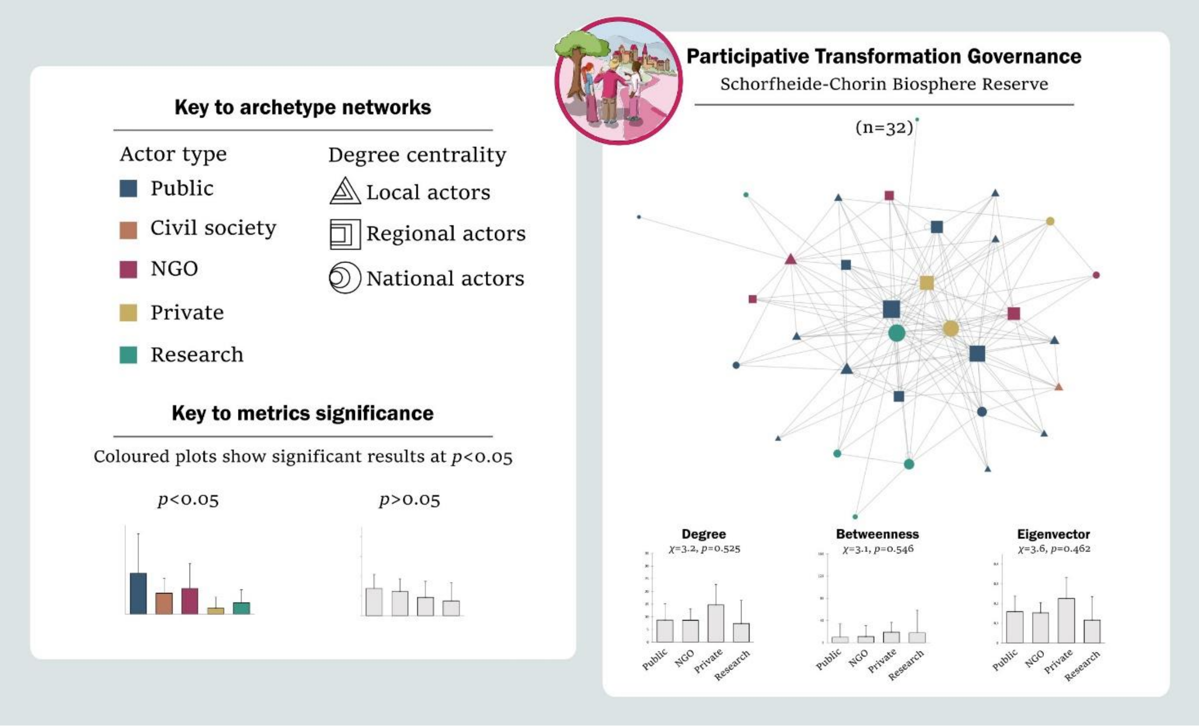

Figure S1. Archetype network of Participative Transformation Governance and bar plots showing centrality metrics (degree, betweenness, eigenvector) of different actor types. The key to archetype networks and the key to metrics significance is common to all following figures depicting archetype networks and centrality metrics.

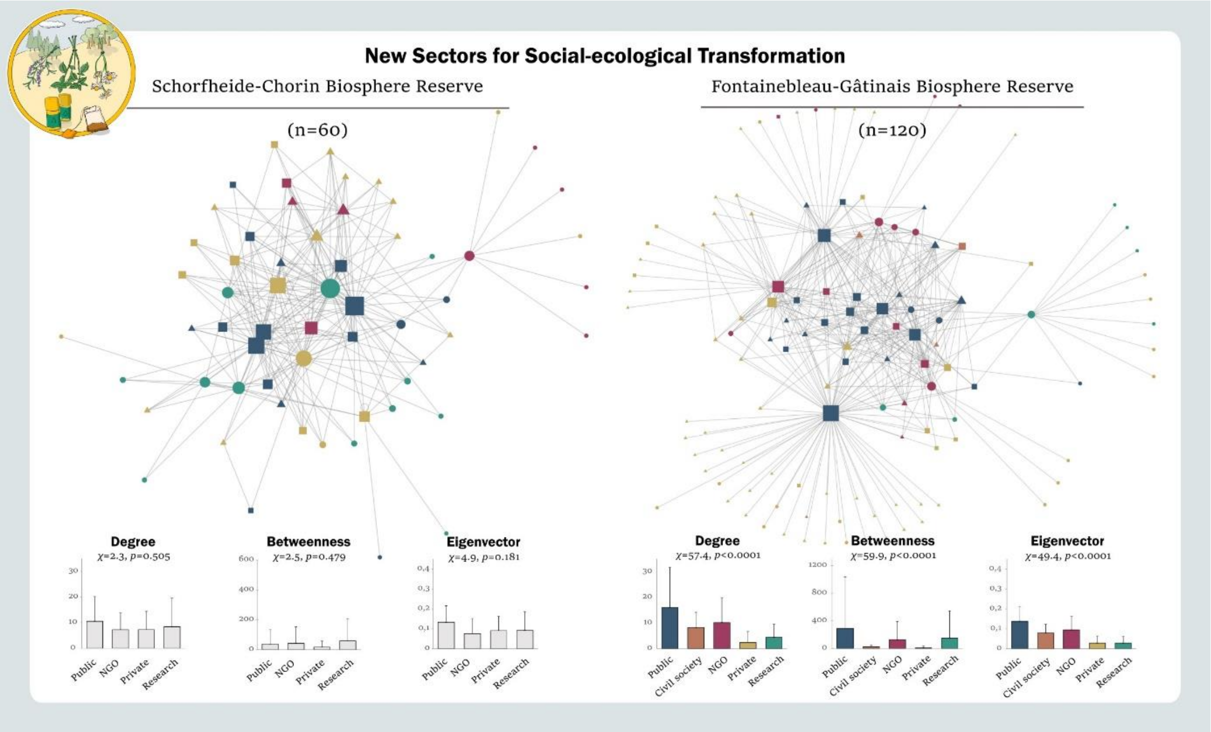

Figure S2. Archetype network of New Sectors for Social-ecological Transformation and bar plots showing centrality metrics (degree, betweenness, eigenvector) of different actor types.

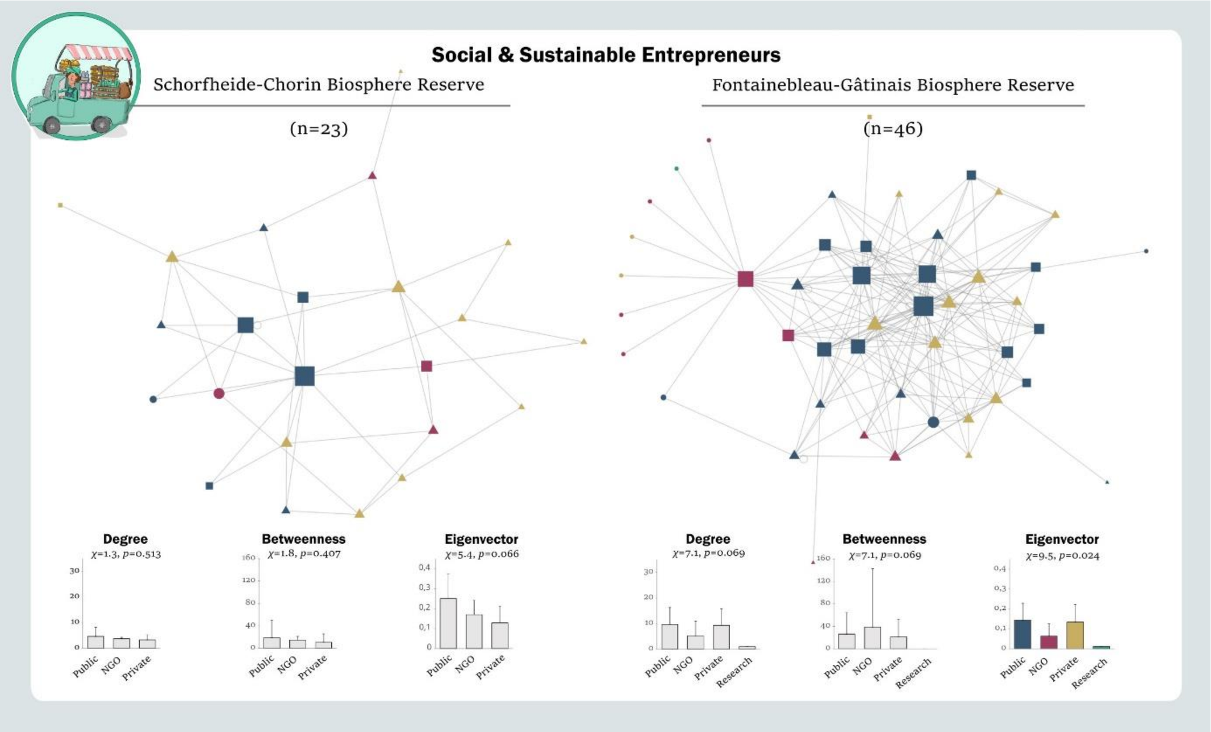

Figure S3. Archetype network of Social and Sustainable Entrepreneurs and bar plots showing centrality metrics (degree, betweenness, eigenvector) of different actor types.

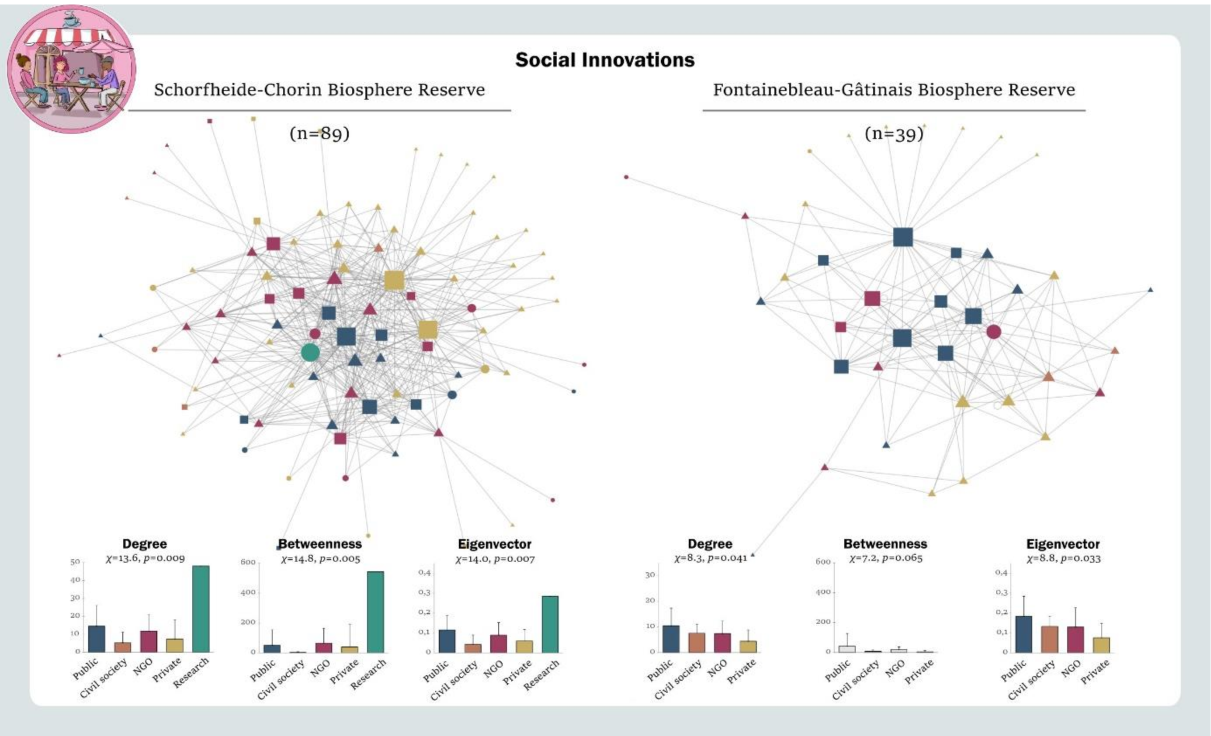

Figure S4. Archetype network of Social Innovations and bar plots showing centrality metrics (degree, betweenness, eigenvector) of different actor types.

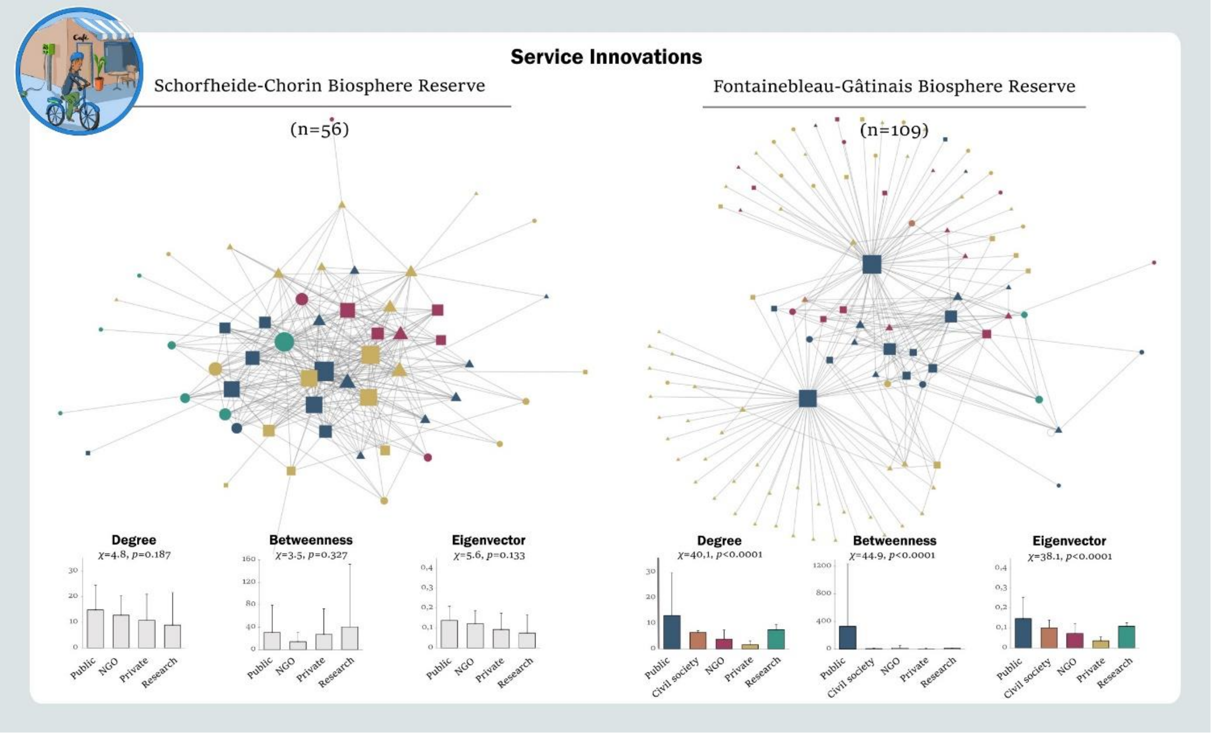

Figure S5. Archetype network of Service Innovations and bar plots showing centrality metrics (degree, betweenness, eigenvector) of different actor types.
